# *STXBP1*-associated neurodevelopmental disorder: a comparative study of behavioural characteristics

**DOI:** 10.1186/s11689-019-9278-9

**Published:** 2019-08-06

**Authors:** Sinéad O’Brien, Elise Ng-Cordell, The DDD Study, Duncan E. Astle, Gaia Scerif, Kate Baker

**Affiliations:** 1 MRC Cognition and Brain Sciences Unit, University of Cambridge, 15 Chaucer Road, Cambridge, CB2 7EF UK; 2 Department of Experimental Psychology, University of Oxford, Anna Watts Building, Radcliffe Observatory Quarter, Woodstock Road, Oxford, OX2 6GG UK; 3 Wellcome Sanger Institute, Wellcome Genome Campus, Hinxton, Cambridgeshire CB10 1SA UK; 4 Department of Medical Genetics, University of Cambridge, Cambridge Biomedical Campus, Hills Road, Cambridge, CB2 0XY UK

**Keywords:** STXBP1, Epilepsy, Intellectual disability, Language, Social

## Abstract

**Background:**

De novo loss of function mutations in *STXBP1* are a relatively common cause of epilepsy and intellectual disability (ID). However, little is known about the types and severities of behavioural features associated with this genetic diagnosis.

**Methods:**

To address this, we collected systematic phenotyping data encompassing neurological, developmental, and behavioural characteristics. Participants were 14 individuals with *STXBP1*-associated neurodevelopmental disorder, ascertained from clinical genetics and neurology services UK-wide. Data was collected via standardised questionnaires administered to parents at home, supplemented by researcher observations. To isolate discriminating phenotypes, the *STXBP1* group was compared to 33 individuals with pathogenic mutations in other ID-associated genes (ID group). To account for the potential impact of global cognitive impairment, a secondary comparison was made to an ability-matched subset of the ID group (low-ability ID group).

**Results:**

The *STXBP1* group demonstrated impairments across all assessed domains. In comparison to the ID group, the *STXBP1* group had more severe global adaptive impairments, fine motor difficulties, and hyperactivity. In comparison to the low-ability ID group, severity of receptive language and social impairments discriminated the *STXBP1* group. A striking feature of the *STXBP1* group, with reference to both comparison groups, was preservation of social motivation.

**Conclusions:**

De novo mutations in *STXBP1* are associated with complex and variable neurodevelopmental impairments. Consistent features, which discriminate this disorder from other monogenic causes of ID, are severe language impairment and difficulties managing social interactions, despite strong social motivation. Future work could explore the physiological mechanisms linking motor, speech, and social development in this disorder. Understanding the developmental emergence of behavioural characteristics can help to focus clinical assessment and management after genetic diagnosis, with the long-term aim of improving outcomes for patients and families.

**Electronic supplementary material:**

The online version of this article (10.1186/s11689-019-9278-9) contains supplementary material, which is available to authorized users.

## Background

It is increasingly possible to identify the genetic cause of severe neurodevelopmental disorders, with potential diagnostic yields now exceeding 50% [1]. For each rare genetic cause, there is an ongoing imperative to characterise associated medical and behavioural features in order to provide evidence-based prognostic advice, targeted family support, and ultimately personalised interventions.

Syntaxin-binding protein 1 (STXBP1, formally known as MUNC18-1) is part of the SEC1 family of membrane-trafficking proteins that interact with SNARE proteins to facilitate the docking of synaptic vesicles at presynaptic active zones to enable neurotransmission [2]. STXBP1 is expressed ubiquitously throughout the brain and neocortex during development and postnatal life. De novo *STXBP1* variants were initially identified in five patients as a cause of Ohtahara syndrome, also known as early infantile epileptic encephalopathy [3]. Thereafter, the epilepsy spectrum associated with *STXBP1* variants expanded to include infantile spasms, West syndrome, Dravet syndrome, Lennox-Gastaut syndrome, and various other types of childhood-onset epilepsy [4–8]. The estimated prevalence of de novo *STXBP1* variants in severe childhood epilepsy is 3% [9]. In parallel, *STXBP1* variants have been discovered in individuals broadly ascertained for neurodevelopmental disorders, with and without epilepsy [7, 10–14]. The prevalence of *STXBP1* variants amongst individuals with unspecified developmental disorders is 0.25–0.5% [15, 16]. Given the 1% prevalence of intellectual disability (ID), the numbers of individuals diagnosed with *STXBP1*-associated neurodevelopmental disorder will rise substantially over the coming years.

Collation of clinical information has highlighted an association between *STXBP1* variants and complex long-term neurodevelopmental impairments, in patients with and without epilepsy. Existing case series suggest that there is considerable variability in the types and severities of neurodevelopmental problems associated with *STXBP1* variants. Reported problems include movement impairments such as ataxia and tremor, language delay and behavioural symptoms including hyperactivity, stereotypies, and autistic features [7, 9, 13, 17–20]. However, despite the increasing number of patients diagnosed with *STXBP1* variants, no study has carried out post-diagnostic, standardised assessments to systematically characterise developmental and behavioural aspects of the *STXBP1*-associated phenotype. These data are necessary to provide families with valid information about the types and severities of neurodevelopmental difficulties that can be expected for an individual diagnosed with an *STXBP1* variant. Hence, the primary aim of the current study was to describe in detail the neurodevelopmental phenotype of children and adolescents with *STXBP1* variants*,* ascertained as broadly as possible.

The secondary aim of the study was to relate neurodevelopmental characteristics associated with *STXBP1* variants to existing basic science literature, including animal models. STXBP1 plays a role in calcium-dependent, short-term synaptic facilitation, which may be particularly relevant to learning [21]. *STXBP1* loss of function mutations lead to broad impairments in synaptic physiology that nonetheless result in specific learning deficits and behavioural features. In cultured neurons from human embryonic stem cells, homozygous mutations impede synaptic transmission which results in neurodegeneration, whereas heterozygous mutations have a milder impact [21]. Homozygous knockout mice have shown that, despite normal brain assembly, lack of *STXBP1* expression inhibits pre-synaptic events which leads to synaptic degeneration, neuronal apoptosis, and early fatality [22–24]. In contrast, heterozygous *STXBP1* mouse models have reduced protein expression and stability, but structurally normal synaptogenesis. Mice demonstrate a seizure phenotype characterised by twitches, jerks, and jumps which often coincided with EEG spike-wave discharges [25]. Currently, there is no systematic, comparative data available on the human behavioural phenotype to verify whether behavioural results in *STXBP1* mouse models [26–28] recapitulate the human disorder. To achieve this, we have compared individuals with *STXBP1* variants to participants with equivalently severe ID, to determine which if any characteristics are consistently observed and are specific to this group. This comparison can add specificity to the prognostic information available to families and clinicians and set hypotheses for future investigation of molecular, neural, and cognitive mechanisms.

## Methods

### Participants and recruitment

Individuals who had previously been diagnosed with a pathogenic de novo variant in *STXBP1* were identified via clinical genetics services, paediatric neurology services, and clinical testing laboratories. Responsible clinicians were provided with a recruitment pack for each potential participant, and 14 families volunteered to participate in the study. Eight participants had been diagnosed with an *STXBP1* variant via the Deciphering Developmental Disorders research study [16], and the remainder had been diagnosed via a clinical testing pathway. For both testing pathways, pathogenicity of variants had been evaluated by the regional genetics service according to ACMG guidelines and local confirmation procedures. The same recruitment procedure was followed for the comparison sample of individuals with neurodevelopmental disorder associated with single nucleotide variants in other ID-associated genes. Participants were recruited with the assistance of 10 regional genetics centres.

### Phenotyping assessments

All 14 participants were assessed in their homes. A standardised, study-specific, structured medical history interview was conducted, followed by the Vineland Adaptive Behaviour Scales, survey interview form [26]. Parents or carers were also given age-appropriate questionnaires to complete which included the Developmental Behavioural Checklist (DBC) [27], Conners-3 short-form [28], Social Responsiveness Scale (SRS-2) [29], and the Movement ABC Parent Checklist [30]. The Movement Assessment Battery for Children (MABC-2) was used to directly assess participants’ manual dexterity, aiming and catching, and balance [30]. Detailed notes and video recordings were obtained in order to document qualitative observations of each participant.

### Data completion rates

For descriptive reporting, data from all *STXBP1* participants are included (*n* = 14) (Additional file 1). For comparative analyses, analysis was restricted to a consistent *STXBP1* group for whom a complete questionnaire dataset was available (‘*STXBP1* full dataset group’, *n* = 8). Partial data collection was achieved for younger participants (protocol only appropriate for ages 4 and above) and for three participants without a fully completed parent questionnaire pack.

### Analysis

Raw scores from questionnaire data were standardised to published normative data. Non-parametric comparisons were conducted throughout (Mann-Whitney *U* statistics).

## Results

### Demographics of *STXBP1* and comparison groups (Table 1)

The *STXBP1* group comprised 14 individuals with a confirmed pathogenic, de novo variant in *STXBP1.* The ID comparison group comprised 33 individuals with confirmed pathogenic or likely pathogenic de novo or inherited variants in other ID-associated genes (Additional file 2). The *STXBP1* full dataset and ID groups did not differ in age, gender or socioeconomic status (SES) distributions. On average, the *STXBP1* group had more severe global adaptive impairments than the ID comparison group (Mann-Whitney *U* = 43.5, *p* = 0.004), with more severe deficits in all adaptive behaviour subscales except for gross motor function (Table 2). To identify specific behavioural characteristics not explained by these global impairments, secondary analyses were carried out between the *STXBP1* full dataset group and a low-ability subset of the ID comparison group, selected to match the range of Vineland Adaptive Behaviour Composite scores. The *STXBP1* full dataset and low-ability ID comparison groups did not differ in age, gender, or SES representation.

**Table 1:** Demographic information

Demographic measure		Group			
		*STXBP1* all (*n* = 14)	*STXBP1* full dataset (*n* = 8)	ID all (*n* = 33)	ID low ability (*n* = 8)
Age	Mean (SD)	9.93 (5.837)	13.63 (2.326)	13.91 (5.553)	14.88 (3.271)
	Range (years)	1–17	10–17	5–25	8–18
Gender	% females(*n*)	71.4 (10)	62.5 (5)	51.5 (17)	62.5 (5)
SES**	Median (SD, range)	6.5 (2.51, 2–10)	7 (1.96, 4–10)	6 (2.33, 1–10)	6.5 (1.98, 3–9)
Epilepsy (ever)	% (*n*)	92.9 (13)	87.5 (7)	39.4 (13)	50 (4)
Epilepsy (current)	% (*n*)	92.3 (12)	85 (6)	62 (8)	75 (3)
Epilepsy age of onset	Mean (SD)	49.67 (67.059)	84.14 (70.115)	70.73 (76.352)	12.67 (9.866)
	Range (months)	1–156	1–156	1–204	6–24
Global intellectual ability	Mean (SD)	45.86 (14.992)	41.0 (12.13)	58.3 (15.661)^#^	48.0 (9.592)
	Range	26–69	31–67	20–96	36–68

**SES calculated using ‘English Indices of Deprivation’ (2015)

^#^Comparison to *STXBP1* full dataset group, Mann-Whitney *U* test, *P* < 0.05

**Table 2:** Adaptive function

Vineland (standard score) Domains Subdomains		Groups			
		*STXBP1* all (*n* = 14)	*STXBP1* full dataset (*n* = 8)	ID all (*n* = 33)	ID low ability (*n* = 8)
Communication	Mean (SD)	42.64 (11.5)	43.38 (12.1)	59.15 (18.6)^#^	47.50 (7.6)
	Range	30–67	33–67	21–100	38–61
Receptive	Mean (SD)	4.82 (2.7)	4.75 (2.6)	8.70 (3.1)^#^	7.13 (1.5)^#^
	Range	2–10	2–10	1–16	5–10
Expressive	Mean (SD)	3.55 (2.7)	3.38 (2.8)	6.88 (4.0)^#^	3.00 (1.9)
	Range	1–8	1–8	1–17	1–6
Written	Mean (SD)	5.55 (1.8)	5.38 (1.8)	7.97 (3.4)^#^	5.88 (1.0)
	Range	4–9	4–9	3–20	5–8
Daily living skills	Mean (SD)	45.57 (19.7)	38.38 (15.5)	57.36 (18.2)^#^	43.00 (13.3)
	Range	25–77	25–73	21–98	30–71
Personal	Mean (SD)	4.71 (4.5)	2.88 (3.8)	6.18 (4.3)^#^	2.75 (2.6)
	Range	1–12	1–12	1–19	1–8
Domestic	Mean (SD)	6.43 (4.3)	4.38 (3.3)	8.73 (3.8)^#^	5.63 (3.3)
	Range	2–13	2–12	3–17	3–13
Community	Mean (SD)	4.86 (3.9)	3.00 (1.9)	6.18 (3.6)^#^	3.88 (2.6)
	Range	1–12	1–7	1–13	1–9
Socialisation	Mean (SD)	52.29 (12.1)	47.75 (8.4)	63.30 (16.5)^#^	56.13 (9.2) ^#^
	Range	34–75	40–67	20–108	46–75
Interpersonal	Mean (SD)	6.00 (3.3)	4.63 (2.1)	7.94 (3.0)^#^	6.63 (1.8)
	Range	2–13	2–8	1–15	4–10
Play and leisure	Mean (SD)	4.93 (3.4)	3.63 (3.2)	7.76 (3.6)^#^	5.00 (3.7)
	Range	1–11	1–11	1–15	1–11
Coping skills	Mean (SD)	6.79 (1.8)	6.00 (1.3)	9.30 (3.1)^#^	8.00 (1.3)^#^
	Range	4–9	5–8	6–20	6–10
Motor	Mean (SD)	60.43 (16.4)	63.25 (17.4)	69.03 (12.4)	62.63 (5.6)
	Range	37–97	40–97	56–107	56–72
Gross	Mean (SD)	8.50 (2.8)	9.63 (3.0)	9.58 (1.8)	9.00 (1.5)
	Range	5–15	6–15	7–16	7–11
Fine	Mean (SD)	8.29 (4.1)	8.00 (3.1)	10.09 (3.0)^#^	8.50 (0.9)
	Range	4–17	4–14	7–20	7–10

^#^Comparison to *STXBP1* full dataset group, Mann-Whitney *U* test, *P* < 0.05

### *STXBP1* group—medical, neurological, and neurodevelopmental characteristics

#### General health

Duration of pregnancy ranged from 32 to 42 weeks. No abnormal ultrasounds or threatened miscarriages were reported. However, five mothers reported persistent vomiting throughout pregnancy. There were concerns about foetal well-being during the birth of six of the participants. Four participants were born by Caesarean section. Only three cases subsequently spent time in SCBU (with none requiring neonatal intensive care). Three individuals were readmitted to hospital within the first month of life for investigation of suspected seizures.

A large proportion of parents (85%) reported feeding issues during infancy and early childhood, common problems being reflux, choking, and difficulty moving solids. Sleep difficulties were reported in four cases during infancy, characterised by problems falling and staying asleep. In two cases, sleep difficulties persisted into adolescence.

Parents also reported a range of sensory issues with regard to hearing, sight, and touch. Six parents reported sight issues including; ptosis, misshapen eye, difficulty focusing, excessive blinking, and long-sightedness. Three parents reported hearing issues: one participant suffered from recurrent ear infections, one had glue ear in both ears, and one participant required hearing aids. A diverse range of sensory issues involving touch and sound were reported in eight cases (3: touch, 3: sound, 2: touch and sound). Three participants enjoyed sensory feedback from sensory objects and people (e.g., hugging and rough play), whereas two did not. One child appeared to enjoy high-pitched sounds such as babies crying, whereas four parents noticed that their children were averse to certain loud sounds such as hoovers, fireworks, and weather. One of these children had received a diagnosis of hyperacusis.

#### Neurological histories

Thirteen *STXBP1* participants have a history of seizures, diagnosed as epilepsy by a paediatric neurologist. The age of seizure onset varied from just after birth to 13 years of age. Half of the *STXBP1* participants (*n* = 7) had a seizure in their first year of life. Four of these participants had a seizure within the first 2 months of life. For the remaining six individuals with epilepsy, the age of onset of seizures was broadly spread across childhood. Three participants experienced their first seizure during the peripubertal phase. The type of seizure also varied. One participant was diagnosed with infant encephalopathy, two had tonic-clonic seizures, and six experienced focal seizures. Seizure phenotypes were mixed or uncertain for the remaining participants. Epilepsy has been treated with a wide range of anti-epileptic drugs (AEDs), the most frequently used was levetiracetam (*n* = 4). Whilst seizure frequency was generally reduced on treatment, no participant was reported to be completely seizure free during the months prior to research assessment. One participant experienced infrequent seizures (approximately three between 5 and 14 years of age) but was not on medication. Four participants also experienced tremors in their hands, which were observed by the researcher during assessments.

#### Developmental milestones

Thirteen participants learned to sit independently from 7 to 42 months. Seven out of thirteen sat independently before 10 months, which is within the normal to mildly delayed range, whilst the remainder were clearly delayed. The youngest child in the sample (1-year-old) had extremely low muscle tone and was not able to sit independently at the time of assessment. Four children did not learn to crawl but ‘bum-shuffled’ or ‘bunny hopped’. Nine participants were walking independently at the time of assessment. However, eight acquired this skill late: between 25 and 60 months old. One participant, aged 14, could walk a few steps but required constant support. Three participants between 1.9 and 6.8 years of age had not yet learned to walk.

Speech and language acquisition were delayed in all cases. Eight participants currently use some verbal communication, with age of first words being between 18 and 156 months (13 years). The age of participants not currently using verbal communication is between 1.1 and 15 years. Of the four participants who can form simple phrases or sentences, three of these acquired this skill late, after 5.5 years of age.

#### Current motor abilities

Vineland gross and fine motor subscale scores reflect the extent of impairment and range of motor abilities within the sample. Gross motor standardised scores ranged between 6 and 15: from severely impaired to average for age (maximum score 24, normative population mean = 15, SD = 3). The participant with the highest score could run, jump, hop, skip, and kick, throw, and catch a ball, whereas, at age 6, the participant with the score was unable to pull themselves to a standing position. Similarly, fine motor standard scores ranged from 4 to 17. Participants with greater fine motor abilities could draw shapes freehand and build three-dimensional structures whereas three participants were unable to turn pages or stack blocks.

The MABC-2 Parent Checklist provided a parent rating of their child’s motor abilities in predictable and unpredictable environments. In addition, a 13-item subscale asks parents to indicate any non-motor factors that may affect their child’s movement. Over 85% of parents in the STXBP1 group reported that being overactive, distractible, impulsive, disorganised, and hesitant contributed to their child’s motor skills difficulties. In addition, 62.5% of children became anxious in stressful, movement-related situations. All participants attempted MABC-2 motor tasks but only one participant was able to complete the full protocol enabling standardised scoring.

#### Current adaptive and communication abilities

The global adaptive function was estimated via the Vineland Adaptive Behaviour Scales (parent interview) composite score. Seven participants had scores in the severe ID range and seven participants were in the mild to moderate ID range. Notably, three participants under 2 years of age scored in the mild ID range, which may indicate increasing diversion from developmental expectations with age.

Vineland Communication standard scores have a mean of 100 and a standard deviation of 15. Participants scored between 33 and 67 (i.e. moderately to severely impaired range). The *STXBP1* participants’ receptive abilities ranged from an inability to listen and attend to speech to being able to recall and carry out instructions given 5 min previously. Expressive ability ranged from difficulty with pre-speech expression to engaging in interactive speech.

#### Current emotional and behavioural difficulties

The Developmental Behavioural Checklist (DBC) was completed by parents to characterise the current behavioural and emotional difficulties they observe in their children (participants over the age of 5). Seven out of eight *STXBP1* participants had a DBC Total Problem Behaviour Score above the clinical cut-off (*T* score 46) indicative of the likely presence of psychopathology. Behaviours such as biting, spitting, ripping things, hitting, and running away from caregivers were common, and parents reported demanding situations and frustration. Two parents remarked that these behaviours had become increasingly difficult to manage following the onset of puberty. High levels of anxiety associated with specific causes such as fireworks, adverse weather, hoovers, lawnmowers, and the emotions of others were reported in five cases (4 females, 1 male). Six parents also reported that their child rarely cried and did not appear to feel pain.

Parents completed the Conners Short Form to assess the characteristics of ADHD and comorbid problems in the sample. The *STXBP1* group showed very elevated scores on the inattention, hyperactivity, learning problems, and peer relations subscales. Two participants had a *T* score in the normal range for executive functions and five participants scored within the normal range on the aggression subscale.

#### Current social functioning

Vineland socialisation subscale scores indicated that the *STXBP1* group demonstrate social adaptive impairments. The Social Responsiveness Scale (SRS-2) was used to characterise atypical social processes linked to autism spectrum features in the sample. Three participants had an SRS total score within the mild to moderately impaired range and five scored above 76, which is indicative of severe social impairment likely to meet criteria for an autism spectrum diagnosis. Participants’ scores fell within the moderate to severe range on social awareness, social cognition, social communication and restricted interests and repetitive behaviours subscales. In contrast, scores on the social motivation subscale were notably preserved and within the normal range. Parents typically described a social profile characterised by self-confidence in social settings and an interest in engaging with peers and adults. The study researchers also observed this strong interest in social interaction, despite limited communication and social skills as assessed by Vineland scales.

### Comparison of behavioural features in the *STXBP1* full dataset and ID groups

#### Motor function

There was no difference in gross motor skills between groups as assessed via Vineland Adaptive Behaviour Scales (Table 2) and Movement ABC (Table 3). However, the *STBXP1* group had poorer Vineland fine motor subscale scores when compared to the ID group (*U* = 74.0, *p* = 0.053). There was no difference between groups in how parents evaluated the contextual factors influencing children’s motor performance.

**Table 3:** Emotional and behavioural characteristics

Outcome measure		Groups		
		*STXBP1* full dataset (*n* = 8)	ID all (*n* = 33)	ID low ability (*n* = 8)
MABC parent checklist				
Motor competence (static environments)	Mean (SD)	24.5 (12.3)	18.45 (9.2)	23.00 (5.6)
	Range	3–42	1–37	11–31
Motor competence (dynamic environments)	Mean (SD)	26.00 (11.9)	22.12 (8.8)	25.5 (6.8)
	Range	3–43	3–39	14–34
Total motor score	Mean (SD)	50.5 (23.6)	40.61 (17.2)	48.5 (10.9)
	Range	6–85	6–74	35–62
Conners (*T* score)				
Inattention	Mean (SD)	84.75 (7.5)	79.19 (12.0)	86.00 (5.3)
	Range	72–92	55–90	79–90
Hyperactivity	Mean (SD)	85.25 (6.5)	72.37 (15.0)^#^	77.25 (13.2)
	Range	73–90	40–90	56–90
Learning problems	Mean (SD)	86.38 (8.3)	79.44 (10.9)	86.13 (5.0)
	Range	66–90	57–90	78–90
Executive functions	Mean (SD)	76 (14.8)	64.56 (14.4)	66.25 (13.3)
	Range	54–90	43–90	48–83
Aggression	Mean (SD)	59.88 (17.4)	52.3 (10.5)	47.5 (4.8)
	Range	44–90	44–83	44–58
Peer relations	Mean (SD)	84.13 (7.2)	80.33 (15.0)	81.38 (16.1)
	Range	72–90	45–90	45–90
DBC (percentile/stratified by ID severity)				
Disruptive	Mean (SD)	68.25 (24.3)	50.91 (28.8)	54.00 (25.2)
	Range	28–90	4–98	26–90
Self-absorbed	Mean (SD)	73.75 (19.2)	70.12 (25.8)	77.75 (17.9)
	Range	34–94	10–100	40–100
Communication	Mean (SD)	75.5 (17.8)	76.42 (23.1)	78.00 (18.8)
	Range	48–96	6–100	40–92
Anxiety	Mean (SD)	42.5 (24.5)	58.3 (26.9)	66.25 (30.1)
	Range	12–76	10–100	10–96
Social relations	Mean (SD)	49.5 (22.8)	58.85 (28.0)	57.00 (27.3)
	Range	24–96	12–100	24–98
Total problems	Mean (SD)	73.50 (24.1)	63.94 (27.4)	67.25 (22.3)
	Range	30–94	0–100	38–98
SRS (*T* score)				
Social awareness	Mean (SD)	78.75 (5.9)	73.64 (11.2)	76.38 (12.3)
	Range	70–87	49–93	56–92
Social cognition	Mean (SD)	73.88 (11.5)	72.7 (11.0)	74.38 (6.6)
	Range	53–92	47–96	67–85
Social communication	Mean (SD)	76.25 (9.2)	72.7 (11.7)	80.88 (11.1)
	Range	66–90	50–96	67–96
Social motivation	Mean (SD)	58.00 (13.6)	64.52 (11.8) ^#^	69.5 (13.6)^#^
	Range	45–89	41–95	58–95
RRB	Mean (SD)	79.00 (11.9)	78.36 (15.2)	87.38 (14.7)
	Range	62–92	48–108	72–108
SRS total score	Mean (SD)	77.75 (9.3)	75.12 (12.0)	81.75 (10.9)
	Range	66–94	49–98	70–98

^#^Comparison to *STXBP1* full dataset group, Mann-Whitney *U* test, *P* < 0.05

#### Emotional and behavioural difficulties

The *STXBP1* and ID groups did not differ on DBC total problem behaviour scores or subscale scores (stratified for ID severity), indicating that emotional and behavioural difficulties in general are not more prevalent or severe in the *STXBP1* group than expected for intellectual disability due to other causes (Table 3). Anxiety and disruptive behaviours were prominent problems for some but not all individuals within the *STXBP1* group; however, group mean DBC anxiety subscale scores did not differ from the ID comparison group. On the Conners scales, the *STXBP1* group were rated as significantly more hyperactive/impulsive compared to the ID group (*U* = 52.0, *p* = 0.026). In summary, the *STXBP1* group demonstrated similar total problem behaviour scores to the ID comparison group, with variation within the group in the severity and types of problems reported for each individual. High hyperactivity/impulsivity scores were more consistent within the *STXBP1* group and on average were more severe than reported for individuals with other monogenic causes of ID.

#### Social functioning

There was a significant difference between *STXBP1* and the ID groups on Vineland Socialisation scores: the ID group on average display stronger skills on all three subscales—interpersonal relations, play and leisure, and coping skills. The groups did not differ on SRS total score or social awareness, social cognition, social communication and restricted interests and repetitive behaviours (RRB) subscales. However, the ID comparison group were more impaired on social motivation, compared to the *STXBP1* group who fell within the ‘normal’ range for this subscale (*U* = 74.0, *p* = 0.06). In summary, the *STXBP1* group demonstrated an atypical profile of social functioning in comparison to ID in general, with more severely impaired everyday social behaviour (on Vineland scales), equivalently impaired social cognition (on SRS), but preserved social motivation.

### Comparison of behavioural features in the *STXBP1* full dataset and low-ability ID groups

**Fig. 1:**
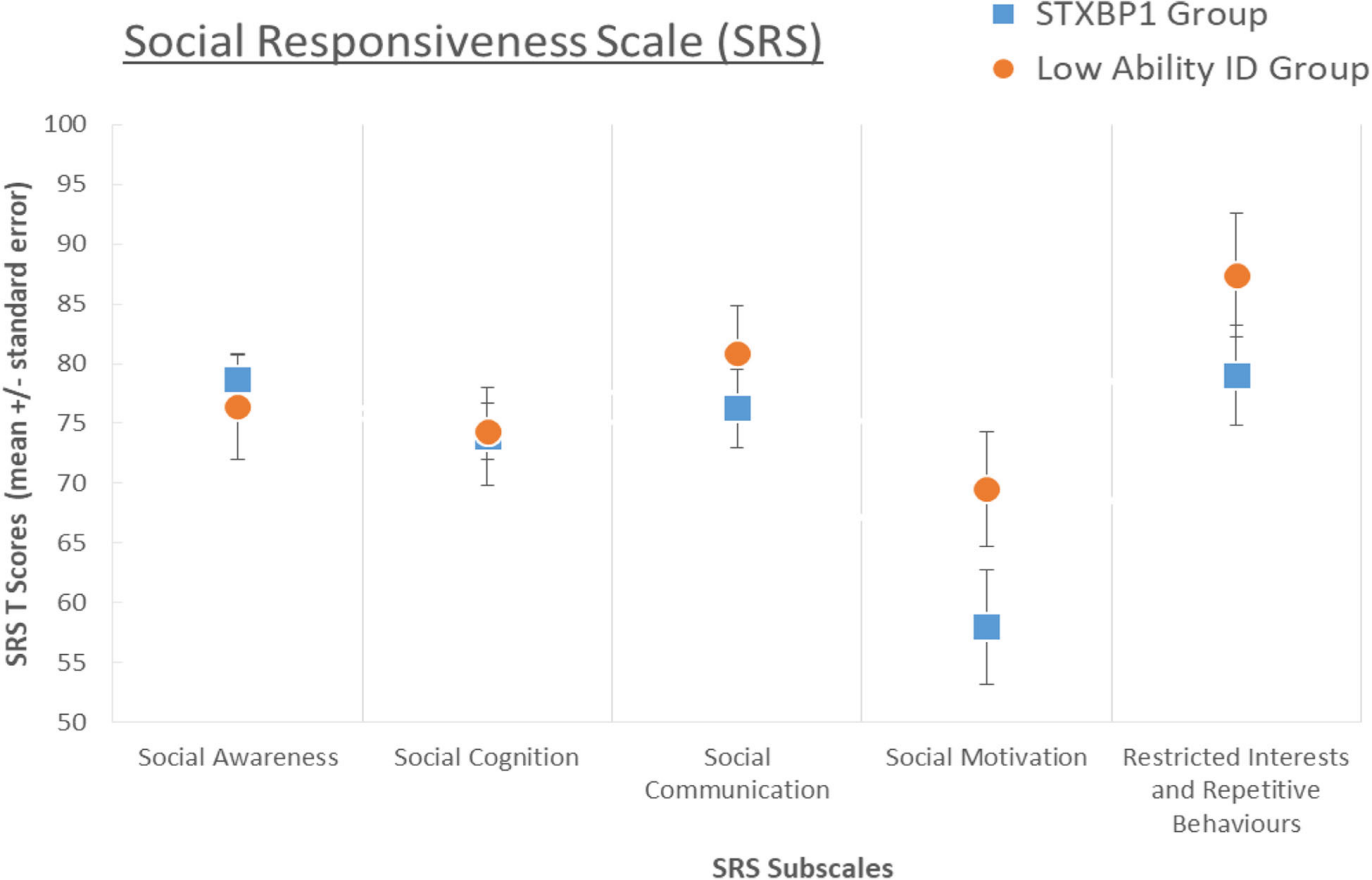
Mean Social Responsiveness Scale subscale *T* scores in the STXBP1 and low-ability ID comparison groups

To explore the specificity of behavioural characteristics associated with *STXBP1* mutation in more detail, secondary comparisons were made to a subset of the ID comparison group selected to match the range of global adaptive abilities within the *STXBP1* group. Although the two groups had comparable global adaptive function scores, communication impairments were more severe in the *STXBP1* group. The comparison group on average demonstrated stronger receptive than expressive abilities whereas the *STXBP1* group showed severe restriction of both receptive and expressive abilities (with significant difference between groups in receptive score: *U* = 11.0, *p* = 0.025). Groups did not differ in gross or fine motor abilities. Behavioural problems assessed via the DBC (total, subscales) did not differ between groups. The *STXBP1* group also did not differ from the low-ability ID group in Conners hyperactivity or impulsivity scores, indicating that although these difficulties are a consistent problem area for the *STXBP1* group, they are not more impaired in these domains than other individuals with equivalent global impairments. Comparison of Vineland scores indicated that everyday social behaviours were more impaired in the *STXBP1* group in comparison to the low-ability ID group (socialisation domain: *U* = 11.0, *p* = 0.027, coping: *U* = 9.0, *p* = 0.014). The analysis of SRS scores (see Fig. 1) indicated a stable pattern of results whether comparing to the whole sample ID group or low-ability ID group—total SRS scores did not differ, social cognitive scores did not differ, restricted and repetitive behaviour scores did not differ, but social motivation was significantly preserved in the *STXBP1* group (*U* = 9.5, *p* = 0.018).

## Discussion

In this study, we systematically assessed the behavioural characteristics of children and adolescents with variants in *STXBP1*, aiming to improve prognostication and management for individuals with this rare genetic diagnosis. Participants in the *STXBP1* group demonstrated complex and persistent difficulties across multiple domains of everyday function and emotional-behavioural development. Consistent problems within the group included severe communication impairments and hyperactivity-impulsivity. Some participants were greatly troubled by anxiety including specific phobia, but these symptoms varied across the group. We observed an additional contrast in some individuals between low social anxiety and high non-social anxiety. In view of moderate to severe ID, it is important to consider whether behavioural characteristics within this group differ from developmental expectations—we found that receptive language ability and socialisation skills (but not hyperactivity) discriminated the *STXBP1* and ID comparison groups, suggesting a specific contribution of aetiology to these features.

We were struck by a consistent profile of social behaviour within the *STXBP1* group. Participants showed great enjoyment of social interactions with family members and demonstrated reciprocal behaviours such as sharing and turn-taking. Researcher experience was that, from the outset of the testing sessions, participants wanted to engage with the researchers (a novel stranger within the participants’ home) particularly in social activities such as drawing, dancing, singing, and playing games. Even participants as young as 2 years held eye contact and were very sociable. On the other hand, parental questionnaire ratings indicated limited understanding of social expectations and difficulties integrating into normal social settings. The observation of significantly stronger social motivation remained true, whether comparing to the whole ID sample or low-ability ID sample, indicating that this feature is not simply a correlate of ID severity. Although several individuals scored above cut-off on the SRS for a likely autism spectrum diagnosis, this profile of social behaviours would not be typical for an individual with autism. These findings support other research into the diverse social developmental trajectories contributing to autism-like impairments in individuals with genomic disorders [31–33].

Epilepsy prevalence within the *STXBP1* group was high, as previously reported, but the type, severity, age of onset, treatment sensitivity, and remission of seizures varied across participants. A relatively high prevalence of focal seizures was reported, in keeping with previous reports [7]. Further studies are needed to examine the contribution of subclinical seizure activity to developmental progress for individuals with *STXBP1* variants, including interactions between low-level sensory processing, cognitive biases, and the social environment to mediate anxieties or prosocial behaviour. In addition, comparison to other groups with similar epilepsy prevalence and seizure characteristics could tease apart aetiology-specific behavioural features from correlates of, for example, focal seizures.

A further goal of this research is to stimulate future investigation of the mechanisms linking presynaptic dysfunction to cognitive and social development, which may ultimately lead to aetiology-specific therapies. Toward this goal, we compared our observations of the *STXBP1* group to published behavioural phenotyping studies of mice with *STXBP1* mutations, to consider consistency across species. The behavioural phenotypes exhibited across different mice models include elevated anxiety, hyperactivity, and impairments in acquiring and maintaining spatial memory and reversal of previously learned strategies [25, 32, 34]. Importantly, not every aspect of learning and behaviour is impaired: *STXBP1* haploinsufficient mice demonstrate normal sociability, preference for social novelty, and normal profiles of attentional control without impulsivity [25]. In one study, social behaviours in *STXBP1* mouse models were examined by introducing a novel mouse into the chamber [27]. Regardless of specific *STXBP1* variant, heterozygous mice spent more time around the novel mouse and displayed normal sociability and preference for social novelty. These findings appear, at least on the surface, to be convergent with our observations of young people with *STXBP1* variants, and future research could explore whether similar neurodevelopmental mechanisms underpin social interactions across species.

In considering potential mechanisms underlying this and other aspects of the *STXBP1* phenotype, we have been struck by the phenotypic similarity with Angelman syndrome (AS). AS is characterised by developmental delay, seizure susceptibility including focal seizures, movement disorders, language deficits, impulsivity, short attention span, and specific phobias [33, 35–38]. The behavioural hallmarks of AS are hyperactivity and hypersociability [34]. Just as in *STXBP1*, individuals with AS display increased social motivation, prolonged social interest, and also excessive smiling and laughing [39]. Socialisation is believed to be underpinned by a network of brain regions including the amygdala, ventral striatum, orbital, and ventromedial regions of the prefrontal cortex. However, different regions and networks may have a greater role in specific aspects of sociability [40]. It has been hypothesised that reciprocal changes within striatal circuits give rise to the atypical social novelty profile associated with AS, consistent with experimental evidence for altered striatal dopamine balance in a mouse model [41, 42]. Examining the striatum and its dependent functional neural systems, in the context of the wider ‘social’ brain network, may provide a starting point for understanding the neural mechanisms driving the atypical social profile in *STXBP1*.

There are several important limitations to this study. The sample size is small and encompasses a wide age range. We aimed to ascertain participants as broadly as possible, from multiple regions of the UK and multiple medical specialties. In comparison to previous studies, the *STXBP1* participant group had a higher proportion of individuals ascertained via clinical genetics rather than paediatric neurology, which may provide a more comprehensive picture of the phenotypic spectrum or may under-represent individuals with early-onset epileptic encephalopathy. The data were collected via parent report, and it was not feasible to corroborate or elaborate via review of medical records. It was also not feasible for researchers collecting data to be blind to genetic diagnoses; however, our use of standardised parent-report questionnaire measures should mitigate potential bias of diagnosis-informed clinician ratings. Non-parametric statistical analyses are included to enhance our descriptive observations and provide an indication of which measures may discriminate between groups. Given the small sample size and large number of measures, adjusting the *p* value threshold for statistical significance to account for multiple comparisons is not practical, and results should be interpreted with due caution. The robustness of our findings will, we hope, be tested in future independent studies with pre-registered hypotheses and predictions building on our exploratory results. In view of small sample size, within-group analysis of behavioural variation was not justified (for example genotype-phenotype correlations, associations with epilepsy severity or age of onset), and these analyses would improve the prognostic utility of our results. A further limitation of the current study is that we have not directly assessed cognitive abilities in young people with *STXBP1* variants and are reliant on parent report of adaptive function and behavioural characteristics. To determine whether specific learning deficits characterise the disorder and contribute to behavioural characteristics, it is necessary to accurately measure relevant aspects of cognition in individuals with moderate to severe ID. However, assessment of cognitive function in individuals with AS or *STXBP1* is challenging due to low levels of understanding and hyperactivity. To achieve this, cognitive tasks must first be developed that assess the capacity for learning different skills (rather than simply documenting deficits), are intuitive, and are developmentally appropriate. In addition to behavioural methods, application of non-invasive physiological approaches such as wireless EEG in semi-naturalistic settings may be feasible and informative. These methods should be developed now and applied in future once a larger number of individuals have been diagnosed via genome-wide testing and can be followed from early childhood through to adult life.

## Conclusion

This research presents the post-diagnostic evaluation of children and adolescents with *STXBP1*-associated neurodevelopmental disorder. Individuals with *STXBP1* variants have a wide range of complex adaptive, social and behavioural characteristics. Via comparison to individuals with other ID-related genetic diagnoses, we have highlighted a more limited set of characteristics which may discriminate individuals with *STXBP1* from individuals with other monogenic causes of neurodevelopmental disorder. Delineating this spectrum should assist in recognising this disorder and supporting families after diagnosis. Moreover, convergence between *STXBP1* phenotypes across species and across disorders (AS) provides a new starting point for understanding the molecular and neural mechanisms which underlie prosocial development.

## Additional files

Additional file 1: STXBP1 Participant Information. (DOCX 17 kb)

Additional file 2: Gene groups and participant numbers recruited to the BINGO study. (DOCX 12 kb)

## Data Availability

All data analysed during this study are included in this published article (and its supplementary information files).

## Abbreviations

ADHD: Attention deficit hyperactivity disorder
AED: Anti-epileptic drug
DBC: Developmental Behavioural Checklist
ID: Intellectual disability
MABC-2: The Movement Assessment Battery for Children 2
SCBU: Special care baby unit
SES: Socioeconomic status
STXBP1: Syntaxin-binding protein 1
