# Supplementary material for: STXBP1-associated neurodevelopmental disorder: a comparative study of behavioural characteristics
Source: J Neurodev Disord. 2019 Aug 6;11:17. doi: 10.1186/s11689-019-9278-9 (PMC6683428; doi:10.1186/s11689-019-9278-9)
Supplement: Supplementary file 1 — STXBP1 Participant Information. (DOCX 17 kb) [file 11689_2019_9278_MOESM1_ESM.docx]

Additional File 1: STXBP1 Participant Information

|  | 1 | 2 | 3 | 4 | 5 | 6 | 7 | 8 | 9 | 10 | 11 | 12 | 13 | 14 |
| --- | --- | --- | --- | --- | --- | --- | --- | --- | --- | --- | --- | --- | --- | --- |
| Decipher ID | - | - | - | 282236 | - | - | 261841 | 258815 | - | 272650 | 265950 | 263903 | 261234 | 260459 |
| Variant | c. 1504_1507dup  p.Ser503Tryfs*23 |  |  | c.364C>T  p.Arg122* | p.Cys354Arg | c.100G>A  p.Asp34Asn | c.1631G>T p.Gly544Val | c.704G>A p.Arg235Gln | c.827dupT | c.533C>T  p.Thr178Ile | c.568C>T p.Arg190Trp | c.1099C>T p.Arg367* | c.778G>T p.Glu260* | c.437del  p.Leu147Trpfs*18 |
| Age (at assessment) | 1.08 | 1.79 | 1.79 | 5.6 | 6.69 | 10.52 | 12.78 | 13.0 | 14.29 | 14.65 | 15.04 | 15.12 | 16.5 | 17.74 |
| Sex | F | F | F | F | M | M | F | F | F | F | F | M | M | F |
| Gestation  (weeks) | 40 | 32 | 32 | 39 | 42 | 39 | 39 | 36 | 40 | 41 | 40 | 41 | 33 | 38 |
| Birth Weight (kgs) | 3.09 | 2.04* | 1.96* | 2.92 | 3.49 | 2.78 | 3.37 | 3.54 | 3.63 | 3.22 | 2.81 | 3.86 | 1.91** | 4.03 |
| ID | Mild | Mild | Mild | Moderate | Severe | Mild | Severe | Moderate | Severe | Moderate | Severe | Severe | Severe | Severe |
| Floppy | Yes | Yes | Yes | Yes | No | No | Yes | Yes | Yes | Yes | No | No | No | Yes |
| Feeding Issues | Difficulty moving solids | Reflux | Reflux | Reflux | Reflux | No | Reflux | Choking and reflux | Reflux | No | Reflux | No | Choking | Choking |
| Sleep Issues | No | No | No | Yes | No | Yes | No | Yes | No | Yes | No | No | No | Yes |
| Sensory Issues | Sight and hearing | Sight | Sight | Sound sensitivity | Ear infections | Touch | Sight, Sound sensitivity | Sight, Sound sensitivity | None reported | Hyperac-usis, Touch | Sight and hearing | Sight | Touch |  |

*Twins

** Participant has a typically developing twin
