# Supplementary material for: STXBP1-associated neurodevelopmental disorder: a comparative study of behavioural characteristics
Source: J Neurodev Disord. 2019 Aug 6;11:17. doi: 10.1186/s11689-019-9278-9 (PMC6683428; doi:10.1186/s11689-019-9278-9)
Supplement: Supplementary file 2 — Gene groups and participant numbers recruited to the BINGO study. (DOCX 12 kb) [file 11689_2019_9278_MOESM2_ESM.docx]

Additional File 2: Gene groups and participant numbers recruited to the BINGO study

| **Genetic Mutations** | **Participant Numbers** |
| --- | --- |
| ARID1B | 5 |
| CASK | 2 |
| CTNNB1 | 1 |
| DDX3X | 10 |
| DLG3 | 2 |
| DYRK1A | 3 |
| EHMT1 | 8 |
| KAT6B | 1 |
| PAK3 | 1 |
| SETD5 | 7 |
| SHANK3 | 3 |
| SHANK1 | 1 |
| SMARCA2 | 1 |
| STXBP1 | 14 |
| TRIO | 2 |
| ZDHHC9 | 1 |
| **Total** | **62** |
